# Improved Mask R-CNN Multi-Target Detection and Segmentation for Autonomous Driving in Complex Scenes

**DOI:** 10.3390/s23083853

**Published:** 2023-04-10

**Authors:** Shuqi Fang, Bin Zhang, Jingyu Hu

**Affiliations:** School of Electronic and Automation, Guilin University of Electronic Technology, Guilin 541004, China

**Keywords:** autonomous driving, environment perception, multi-target, Mask R-CNN, ResNeXt, efficient channel attention module, FPN, CIoU

## Abstract

Vision-based target detection and segmentation has been an important research content for environment perception in autonomous driving, but the mainstream target detection and segmentation algorithms have the problems of low detection accuracy and poor mask segmentation quality for multi-target detection and segmentation in complex traffic scenes. To address this problem, this paper improved the Mask R-CNN by replacing the backbone network ResNet with the ResNeXt network with group convolution to further improve the feature extraction capability of the model. Furthermore, a bottom-up path enhancement strategy was added to the Feature Pyramid Network (FPN) to achieve feature fusion, while an efficient channel attention module (ECA) was added to the backbone feature extraction network to optimize the high-level low resolution semantic information graph. Finally, the bounding box regression loss function smooth L1 loss was replaced by CIoU loss to speed up the model convergence and minimize the error. The experimental results showed that the improved Mask R-CNN algorithm achieved 62.62% mAP for target detection and 57.58% mAP for segmentation accuracy on the publicly available CityScapes autonomous driving dataset, which were 4.73% and 3.96%% better than the original Mask R-CNN algorithm, respectively. The migration experiments showed that it has good detection and segmentation effects in each traffic scenario of the publicly available BDD autonomous driving dataset.

## 1. Introduction

With the rapid development of deep learning and the advancement of intelligent control technology, autonomous driving is gradually becoming the focus of research in various fields. For autonomous driving, three key technologies need to be improved, namely, perception, decision making, and execution [1]. Of these technologies, environment perception is considered to be the eyes of the self-driving vehicle and is the basis and prerequisite for behavioral decisions and executive control. The environment perception of self-driving vehicles on the road with the help of cameras can be seen as the detection and segmentation of multiple targets in front of the vehicle. Therefore, deep learning-based target detection algorithms and instance segmentation algorithms can be applied to environment perception in autonomous driving to help self-driving vehicles obtain information about their surroundings through visual information [2].

Based on the network structure as well as the detection principle, deep learning-based target detection algorithms can be divided into one-stage and two-stage target detection methods. One-stage target detection algorithms achieve higher prediction speed, while two-stage target detection algorithms have region proposal steps with higher localization and object recognition accuracy [3]. In 2016, Redmon et al. proposed a one-stage target detection framework, YOLO, which transforms the target detection problem into a regression problem to realize fast real-time target detection [4]. Liu W et al. introduced multi-scale detection and proposed the single shot multibox detector (SSD) [5]. By detecting multiple targets at different scales through different layers of the neural network to suggest the accuracy of small targets, two-stage target detection divides target detection into two parts, namely, one for proposal generation and one for target classification within the proposal. Girshick et al. proposed R-CNN in 2014, which became the basis of research on two-stage target detection algorithms [6]. By generating proposals through selective searching, the images are then classified by support vector machines. Kai-Ming He et al. optimized R-CNN for speedup and proposed SPPNet by introducing the spatial pyramid pooling model in 2015 [7]. Girshick et al. propose Fast R-CNN on the basis of R-CNN [8], and later with Kai-Ming He proposed Faster R-CNN, which becomes a two-stage target detection model with detection speed closest to real-time in the R-CNN family [9].

Instance segmentation algorithms are different compared to other algorithms in the field of computer vision such as target detection, image classification, etc. Instance segmentation can be seen as labeling out each instance in an image with the aim of labeling out its instances for each pixel. Bai et al. used FCN lattices to learn the energy of the watershed transform and segment the image into several regions by energy segmentation [10]. Gao et al. used pixel-pair affinity pyramids to learn the probability of two pixels belonging to the same instance and generate instances sequentially by cascaded graph partitioning [11]. In 2015, Dai et al. proposed an instance segmentation model using a cascade structure of shared convolutional features by breaking the instance segmentation into three different subtasks to achieve a better effect of instance segmentation [12]. In 2017, Kai-Ming He et al. proposed Mask R-CNN, which achieves instance segmentation by adding a parallel third mask output through an extension for Faster R-CNN [13]. Yi Li et al. presented the first fully convolutional end-to-end solution for instance-aware semantic segmentation tasks. It inherits the merits of FCNs for semantic segmentation and instance mask proposals [14]. Daniel Bolya et al. proposed YOLACT in 2019, with the main idea of directly adding a mask branch to the one-stage target detection algorithm without adding any RoI pooling operation to split the instance segmentation into two parallel branches [15]. In 2020, Wang et al. proposed SOLO, which introduces instance categories and enables direct instance segmentation by quantifying target centers and target sizes [16]. In 2021, Lei Ke et al. proposed BCNet, a two-layer instance segmentation network under masking perception, to explicitly model the masking relationship using a two-layer structure that naturally decouples the boundaries of occluded instances [17]. The interactions during mask regression between them are considered, and the interactions during mask regression between them are considered. In 2022, Tao Zhang et al. proposed a new contour-based image instance segmentation algorithm, E2EC, for high-quality segmentation [18]. Their use of a learnable contour initialization architecture with a multi-directional alignment label sampling method allows the E2EC model to exhibit more advanced segmentation performance. In 2023, He et al. proposed Fastlnst, a query-based real-time instance segmentation model, which mainly includes real-time activation-guided query, dual-path update strategy, and true mask-guided learning for better segmentation performance [19]. In 2023, Zhang et al. proposed a mask-piloted Transformer MP-Former, which additionally adds real masks with noise to the mask attention and trains the model to reconstruct the original masks [20]. It effectively improves the mask prediction accuracy and significantly optimizes the model performance.

Although Mask R-CNN shows good instance segmentation effect, it still has some shortcomings because it does not have good generalization ability when applied to complex traffic scenes. To address the shortcomings of Mask R-CNN when it is difficult to perform instance segmentation and localization for dense multiple targets in autonomous driving scenarios, this paper improved on the basis of Mask R-CNN to adapt it to environment perception under autonomous driving.

## 2. Mask R-CNN Instance Segmentation Algorithm Model

The detection of targets such as vehicles, pedestrians, and non-motorized vehicles is one of the core problems of environment perception in self-driving cars. Most traffic accidents are caused by the collision of vehicles, pedestrians, and non-motorized vehicles, so the detection and segmentation of such objects in the traffic scene is helpful to avoid the occurrence of traffic accidents [21]. As a two-stage instance segmentation model, Mask R-CNN extends Faster R-CNN and adds a parallel mask branch to achieve end-to-end multi-target detection and segmentation.

### 2.1. The Model Structure of Mask R-CNN

**Figure 1:**
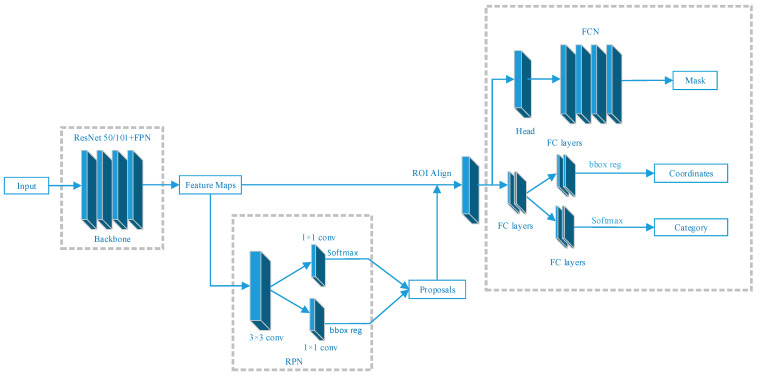
Mask R-CNN model structure. Adapted from Ref. [22].

Mask R-CNN is based on the Faster R-CNN with some improvements, and the structure is shown in Figure 1. The backbone of the feature extraction network is changed from VGG to ResNet and combined with the structure of the Feature Pyramid Network (FPN) to add multi-scale information while solving network degradation. The pooling of the region of interest uses Region of Interesting Align (RoI Align) to replace RoI Pooling. The pooling of the region of interest using bilinear interpolation solved the quantization mismatch problem caused by rounding off during pooling and improves the localization accuracy. It also generates a high-quality mask for detecting objects, which can obtain a perfect effect of segmentation.

Mask R-CNN uses a two-stage target detection method. In the first stage, the proposals are obtained through the Region Proposal Network (RPN). In the second stage, the features of target locations are obtained based on the proposal and regions of interest generated in the first stage, and finally classification, localization, and mask prediction are performed.

### 2.2. Backbone Feature Extraction Network

**Figure 2:**
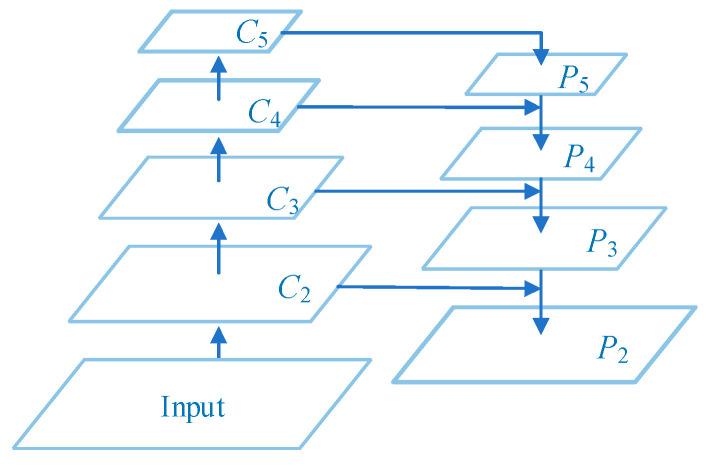
Backbone feature extraction network. Adapted from Ref. [24].

The feature extraction network of Mask R-CNN is divided into two paths: bottom-up and top-down. The bottom-up path is a module composed of residual structures of different sizes, which is responsible for feature extraction of the image. Taking ResNet-101 as an example, as shown in Figure 2, the residual modules are noted as C1, C2, C3, C4, and C5. The output of each module contains different levels of feature information, and the size of the mapping map output by the modules is halved from the bottom-up module by module through the residual structure and the convolution with step size of 2 between modules [23]. The top-down path fuses the abstract features of high-level semantic information with low-level detailed features. The high-level feature maps are reduced to the resolution corresponding to the bottom-up branched feature maps by 2-fold upsampling. The new feature maps P2, P3, P4, and P5 are obtained by adding them pixel by pixel and convolving 3 × 3 to make the high-level semantic information better represented in the mapped images.

### 2.3. Regional Proposal Network—RPN

After the input images are extracted by the backbone feature extraction network, a series of feature maps is obtained. These feature maps are then sent into RPN, which performs preliminary classification and prediction on them, predicting the presence or absence of targets as well as bounding box regression prediction. Here the preliminary RoIs are obtained, and the RoIs include the bounding box regression values of the region of interest in the original image.

**Figure 3:**
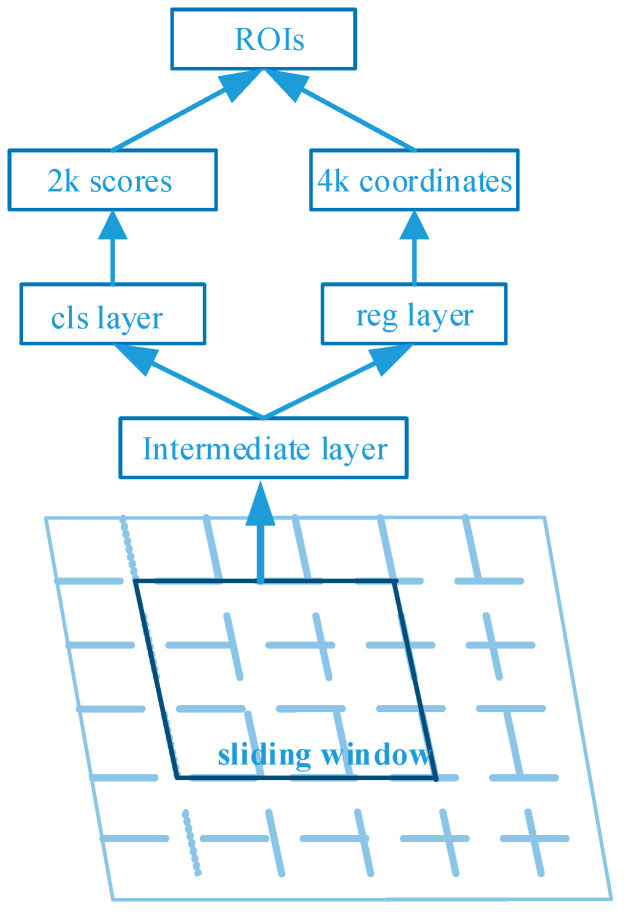
Principle of anchor generation. Adapted from Ref. [9].

The RPN network takes anchors on the feature map to calculate the positions of proposals, and the principle of anchor generation is shown in Figure 3. A sliding window of n × n is set on the feature map to generate k identically centered anchors corresponding to each point in the feature map. To fit various sizes of targets, the anchor is set to 3 area sizes as well as 3 aspect ratios, for a total of 9 sizes; thus *k* = 9. Then, the sliding window is used to convolute the feature map, and the classification layer and regression layer composed of a fully connected network are respectively input through the middle layer to describe the position and type of information of the anchor. The classification layer outputs 2*k* fore-and-aft probability values, and the regression layer outputs 4*k* coordinate values. The RPN corrects the length, width, and center of each anchor based on the correction values calculated by the regression, and the corrected RoIs are sent to the subsequent network after filtering.

### 2.4. RoI Align

**Figure 4:**
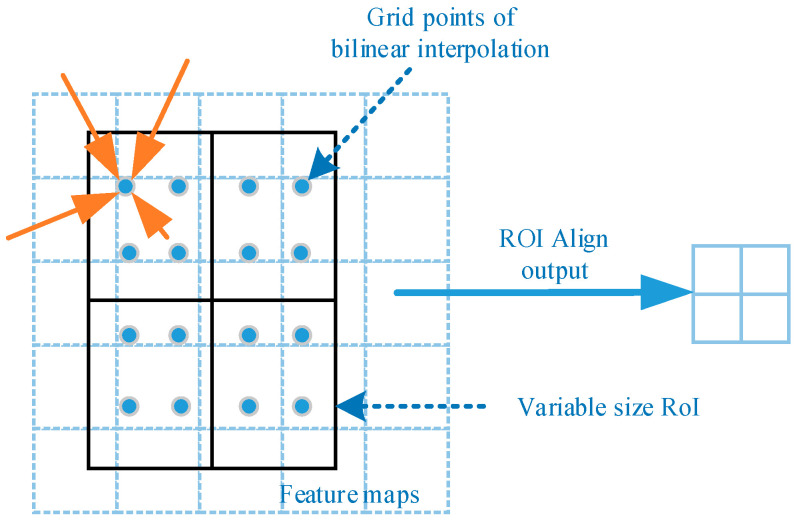
Bilinear interpolation. Adapted from Ref. [13]. The orange arrow points to the center point obtained after the region has been quadratically divided.

RoI Align serves to pool the corresponding regions in the feature maps into fixed-size feature maps based on the location coordinates of the RoIs for subsequent classification and regression operations. The whole feature aggregation process is transformed into a continuous operation by canceling the quantization operation and using bilinear interpolation [25] to obtain the values on the pixel points with floating point coordinates. The operation process is as follows:

RoI Align crops the candidate regions from the feature maps and pools them into 7 × 7 and 14 × 14 feature maps. These two sizes are the specified input sizes for the target classification, and localization and mask generation stages, respectively, as shown in Figure 4. The region proposals are first kept floating-point without quantization. Then it is partitioned into *k × k* cells with no quantization of edges. Finally, each cell is quadratically divided, and four centers are found. The coordinate values of these four locations are calculated by bilinear interpolation, and maximum pooling is performed. RoI Align thus transforms the whole feature aggregation process from a discrete to a continuous operation, solving the problem of errors caused by mismatched quantization twice in the RoI Pooling operation.

### 2.5. Mask Branch

Mask R-CNN adds the “head” part after RoI Align in order to make the predicted masks more accurate, thus expanding the output dimension of RoI Align to achieve more accurate predicted masks. In the training session of the mask branch, k mask prediction maps are output (one for each class), and average binary cross-entropy loss is used for training. The mask branch uses a fully connected layer for each RoI with a segmentation output dimension of k × m × m: a binary mask of m × m for k categories. The use of RoI Align in Mask R-CNN ensures that RoIs can be aligned when mapped to the original map, so that pixel-to-pixel operations can be accomplished, and pixel-level alignment errors can be reduced.

## 3. Improved Mask R-CNN Model

Mask R-CNN can achieve better detection and segmentation of targets, but there are still the following main problems for the detection and segmentation of multiple targets in complex traffic scenes:

(1) In urban traffic scenarios, there are multiple targets in front of the car, such as cars, pedestrians, trucks, and so on. When there is traffic congestion or a large number of pedestrians in front, the problem of overlapping targets is likely to occur. This will lead to individual targets in front of the car not being accurately detected and segmented.
(2) In complicated and variable weather, the detection and segmentation of targets are easily affected by light and weather, which leads to false detection or low detection accuracy.

**Figure 5:**
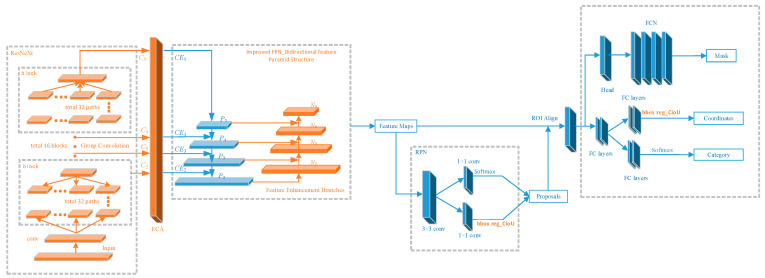
Improved Mask R-CNN model structure: the orange part shows the improvement points proposed in this paper.

In this paper, we improved on the Mask R-CNN model to make it more suitable for multi-target detection and segmentation in complex traffic scenes. The improved network model is shown in Figure 5.

In the ResNeXt section, the input image is first passed through a convolution kernel of size 7 * 7 with a convolutional stride of 2 for the conv operation. The input image is fixed as a feature layer of size 112 * 112 * 64. Then it enters the first block module, in which group convolution is used to increase the diagonal correlation between the convolution kernels and reduce their training parameters. A total of 32 groups are divided to do the convolution operation on the input separately, and then the output of the 32 groups is combined and used as the input of the next block. There are 16 block modules in ResNeXt, divided into four parts. The first part contains 3 blocks with 112 * 112 * 64 inputs and 56 * 56 * 256 outputs. The second part contains 4 blocks with 56 * 56 * 256 inputs and 28 * 28 * 512 outputs. The third part contains 6 blocks with 28 * 28 * 512 inputs and 14 * 14 * 1024 outputs. The fourth part contains 3 blocks with 14 * 14 * 1024 inputs and 7 * 7 * 2048 outputs. The outputs of four components are obtained by ResNeXt: C2, C3, C4, and C5. They are input into the ECA attention mechanism module for each channel weight allocation to obtain CE2, CE3, CE4, and CE5 and input into the improved FPN structure.

CE2–CE5 are fused with the features generated by the top-down layer structure based on the output of the CE5 layer through a lateral connection to obtain four fused multi-scale features of P2–P5, with the width or height of adjacent feature layers differing by a factor of 2. CE2–CE5 are fused with the features generated by the top-down layer structure based on the output of the CE5 layer through a lateral connection to obtain four fused multi-scale features of P2–P5, with the width or height of adjacent feature layers differing by a factor of 2. In addition, a bottom-up feature enhancement branch is added. First, the bottom features are convolved by a convolutional layer with a convolutional step size of 2 and convolutional kernel size of 3 * 3 to change the width and height of the input feature map to half of the original one. Next, a nonlinear layer (Relu [26]) is adopted to reduce the problem of overfitting. Then, the top layer features are passed through a convolutional layer with a convolutional step of 1 and convolutional kernel of 1 * 1 to match the number of channels of the bottom layer features. Finally, the Ni and Pi + 1 layers are summed by elements, and the result is passed through a convolutional layer with a convolution step of 1 and convolutional kernel size of 3 * 3 to obtain the new fused features N2–N5.

The feature maps obtained after ResNeXt and the improved FPN network are sent to the RPN network. The RPN network performs preliminary classification prediction and bounding-box regression prediction on the feature map and then obtains preliminary RoIs, where the loss function of bounding-box regression prediction is CIoU. A large number of RoIs is obtained after the classification prediction and bounding-box regression prediction of the RPN network, and each RoI contains its coordinate position in the original map. The feature map is pooled into a fixed size of k * k by RoI Align to fit the size requirement of the feature map in the final classification prediction and bounding-box regression prediction of the model.

The fixed size feature maps are obtained in RoI Align, and each target corresponds to one feature map. These feature maps are sent to the fully connected layer for final classification prediction and bounding-box regression prediction, where the loss function for bounding-box regression prediction is CIoU. Finally, the feature maps with targets are input into the FCN network to obtain the final mask information of the targets and to perform pixel-by-pixel prediction [27].

### 3.1. ResNet Backbone Network Improvements

In order to balance the model complexity and model accuracy, ResNeXt-50 is adopted as the backbone network in this paper.

**Figure 6:**
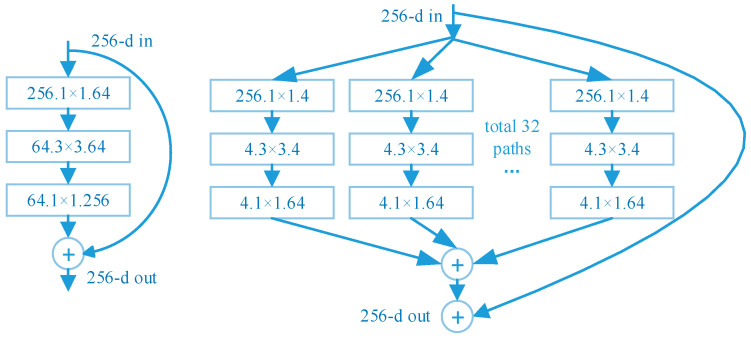
ResNet (**left**) and ResNeXt (**right**).

The ResNeXt network proposes a parallel stacking of blocks with the same topology instead of the original ResNet block with three convolutional layers [28]. The concept of cardinality is introduced in the block, which refers to the number of identical branches in a block. The accuracy of the model is improved without significantly increasing the number of parameters, and the hyperparameters are also reduced due to the same topology, which makes it easier to port the model. As shown in Figure 6, the ResNet network structure is shown on the left and the ResNeXt structure is shown on the right.

Unlike Resnet, ResneXt first groups the input feature maps into 32 groups and downscales them with a 1 * 1 convolution operation. Then it performs a convolution operation with a convolution kernel size of 3 * 3 for each group of branches, and finally it up-dimensions them by a 1 * 1 convolution operation in which the grouped convolution can reduce the computation and parameter amount of the network to 1/g of the normal convolution with the same input and output, where g is the number of groupings. However, to improve the feature extraction effect of the backbone network, ResNeXt increases the number of feature layer channels per branch after grouping. Finally, the number of network parameters of Resnet-50 and ResNeXt-50 is kept almost the same, but the feature extraction effect of the backbone network is effectively improved.

### 3.2. Feature Pyramid Network Improvements

**Figure 7:**
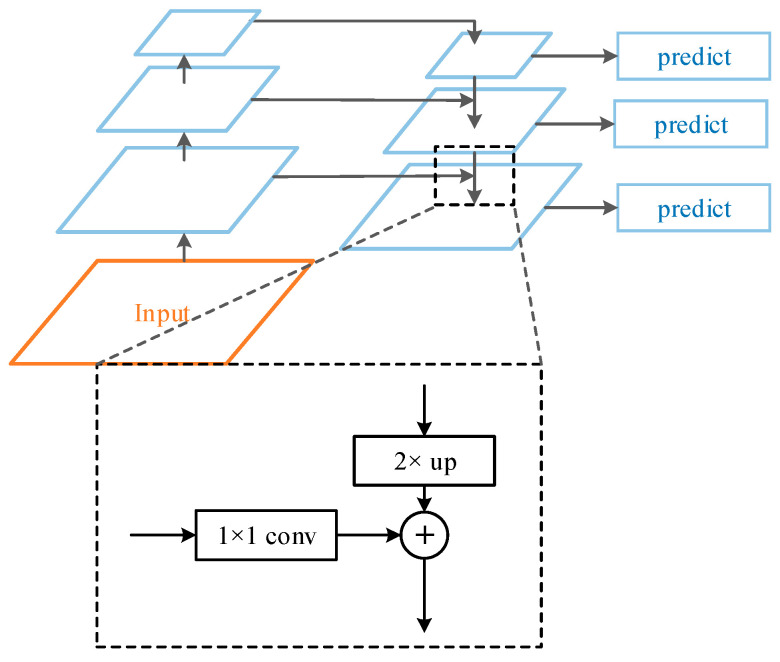
Feature Pyramid Network topology. Adapted from Ref. [24].

Feature Pyramid Network (FPN) is a network topology that aggregates higher-level features with lower-level features, allowing the model to learn information that retains both location and stronger semantic information [24]. Its network topology is shown in Figure 7. It can be observed that the FPNs are predicted by up-sampling the higher-level feature maps and then superimposing them horizontally with the semantic information at lower levels than them. This results in a feature map with strong semantic information and thus achieves the effect of feature fusion.

**Figure 8:**
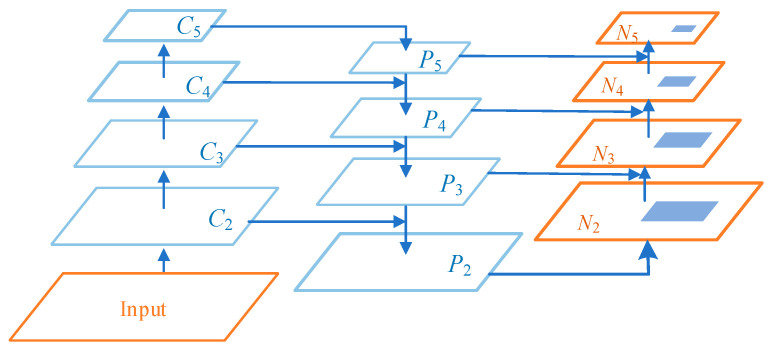
Improved FPN structure. Adapted from Ref. [29].

Influenced by the FPN, Shu Liu et al. proposed a path aggregation network called Path Aggregation Network PANet and achieved excellent results [29], whose backbone feature extraction framework is shown in Figure 8. From the figure, we can see that PANet improves the structure of FPN by adopting a top-down and bottom-up bidirectional feature pyramid fusion structure. The bottom-up path enhancement strategy is added on the basis of the original FPN top-down horizontal stacking combined with downsampling, which aims to shorten the information path and utilize the shallow-level accurate localization, effectively improving the utilization of the underlying features of the network.

The improved FPN structure provides a new propagation path for the delivery of the underlying features through the lateral connections. Compared with the original propagation method of passing through hundreds of layers of the backbone network to the top layer, this multi-path network information flow aggregation method avoids the risk of information loss caused by the bottom layer features through the backbone network. It shortens the information path between the bottom layer and the top layer, solves the problem that the top layer feature map of FPN cannot effectively contain the bottom layer localization information, and enhances the utilization of the bottom layer feature information.

### 3.3. Efficient Channel Attention Module ECA

In the inference process of the Mask R-CNN algorithm, the recognition of medium and large targets is conducted by the high-level feature maps, which are also subject to targeting missed detection due to its low output resolution. In this paper, we introduced the ECA module to optimize the low-resolution semantic information map at a high level of output of the Mask R-CNN algorithm and then obtain the information map with low-resolution and high semantic features to better identify medium and large targets [30] so as to achieve the mean Average Precision (mAP) improvement.

**Figure 9:**
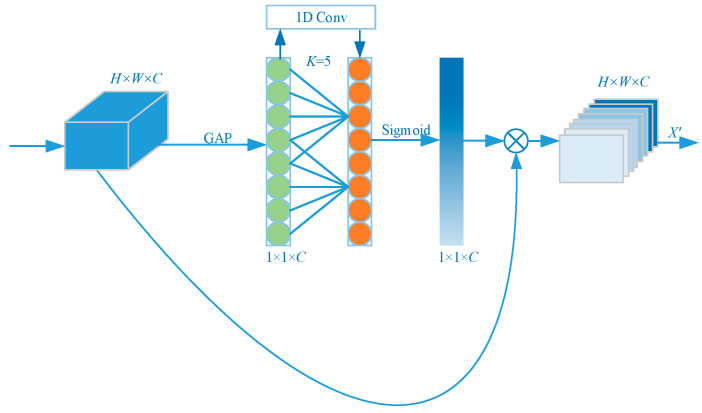
Structure of the Efficiency Channel Attention module. Adapted from Ref. [30].

The attention mechanism is a signal processing mechanism that mimics the human brain, and the strategy is well adapted and gainful for computer vision tasks [31]. The ECA structure is also used to obtain the importance of different feature channels by means of supervised learning. The nonlinear fully connected layer in SENet is improved to avoid the effect of the dimensionality reduction operation on channel attention and to ensure the efficiency and computational effectiveness of the network [32]. The module does not require dimensionality reduction to achieve cross-channel interaction; after the global averaging pooling operation on the output feature map, a one-dimensional convolution operation with convolution kernel size k is used to achieve the interaction of different channel information [33]. The ECA module topology is shown in Figure 9.

The relevance of the feature channels can be expressed as follows:

(1) $$\omega =\sigma \left(CID_{k}\left(y\right)\right)$$

where *CID* denotes a one-dimensional convolution operation, y represents the input weight value, and σ represents the sigmoid function. The parameter k represents the size of the convolution kernel in the one-dimensional convolution, and the value of k is mapped to the number of channels, C, which can be expressed as

(2) $$k=\psi \left(C\right)={\left|\frac{\log_{2}\left(C\right)}{\gamma }+\frac{b}{\gamma }\right|}_{odd}$$

where k denotes the convolution kernel size, C is the number of channels, and odd is the nearest odd number taken. Parameters γ and b are set to 2 and 1, respectively, for changing the ratio between the number of channels C and the convolution kernel size k.

### 3.4. Optimization of Loss Functions

The bounding-box regression loss function smooth L1 loss is replaced with the CIoU loss function to speed up model convergence.

In the entire network structure, there are five predictors. The loss functions corresponding to these five predictors are the classification result $L_{rc}$ of the RPN, the bounding box regression prediction $L_{rb}$ of the RPN, the final classification result $L_{cls}$, the final bounding box regression prediction $L_{box}$, and the final mask image prediction $L_{mask}$, respectively. The combination of the above loss functions gives $L_{}$:

(3) $$L=L_{rc}+L_{rb}+L_{cls}+L_{box}+L_{mask}$$

where the bounding-box regression prediction $L_{rb}$ for RPN and the final bounding-box regression prediction $L_{box}$ are calculated using the smooth L1 loss function as follows:

(4) $$L_{rb}=\sum_{i\in x,y,w,h}smooth({\overset{⌢}{G}}_{i}-p_{i})$$

(5) $$L_{box}=\sum_{i\in x,y,w,h}smooth({\overset{⌢}{G}}_{i}-p_{i})$$

(6) $$smoothL_{1}(x)=\left\{\begin{array}{cc} 0.5x^{2} & if\left|x\right|<1\\ \left|x\right|-0.5 & otherwise \end{array}\right.$$

where ${\overset{⌢}{G}}_{i}$ is the position (coordinates and dimensions) of the ground truth box, $p_{i}$ is the position (coordinates and dimensions) of the prediction box, and x is the difference between the ground truth box and the prediction box.

When smooth L1 loss is used to calculate the bounding box loss for target detection, the losses of the four points are derived independently and then summed to obtain the final bounding box loss [8]. The premise of this approach is that the four points are independent of each other, but there is actually some correlation. Furthermore, evaluating the metrics of the detection box is carried out using IoU, which is not equivalent to the loss. Multiple detection boxes may have the same loss, but the IoU may vary greatly. In order to solve this problem, CIoU loss is introduced as the loss function of the bounding box regression algorithm in this paper. CIoU loss takes the distance, overlap rate, and scale between the ground truth box and the prediction box as the judging criteria. Moreover, the penalty term is added to improve the target regression accuracy, and the loss value converges faster [34]. The CIoU is defined as follows:

(7) $$CIoU=IoU-\frac{\rho^{2}(b,b^{gt})}{c^{2}}-\alpha v$$

where b is the center of the prediction box, $b^{gt}$ is the center of the ground truth box, ρ is the Euclidean distance between the two centroids, $c^{2}$ is the area of the smallest enclosed area that can contain both the prediction box and the ground truth box, v is the weighting factor, and α is the similarity ratio used to measure the length and width. Parameters α and v are defined as follows:

(8) $$\left\{\begin{array}{c} a=\frac{v}{1-IoU+v}\\ v=\frac{4}{\pi^{2}}{\left(\arctan\frac{w^{gt}}{h^{gt}}-\arctan\frac{w}{h}\right)}^{2} \end{array}\right.$$

The CIoU bounding box loss function is defined as follows:

(9) $$L_{CIoU}=1-IoU+\frac{\rho^{2}(b,b^{gt})}{c^{2}}+av$$

It can be seen that the advantage of the CIoU loss function used in this paper is that the loss factors such as overlap area, centroid distance and aspect ratio, and other bounding box regressions are taken into account, which makes the model converge faster and with higher accuracy and will be more reasonable and flexible in optimizing the network error.

## 4. Experimental Results and Discussion

### 4.1. Experimental Environment Configuration

This experimental training platform used a desktop computer with a Windows 10 operating system, and the main hardware configuration was as follows: AMD Ryzen7 5700X CPU, NVIDIA GeForce GTX 3080Ti. The Mask R-CNN algorithm was implemented using the deep learning framework Pytorch 1.13.0 and Python 3.8.

### 4.2. Dataset

The COCO dataset is a general dataset for studying instance segmentation [35], but there is fewer data for road environments, which is not suitable for relevant application scenarios such as autonomous driving. The datasets that provide segmentation data for road environments currently include KITTI, CityScapes, Apllo, etc. The CityScapes dataset contains a large number of complex street view data with good data quality, which is suitable for the application scenario of this study [36]. Therefore, the CityScapes dataset was chosen for model training in this study.

**Figure 10:**
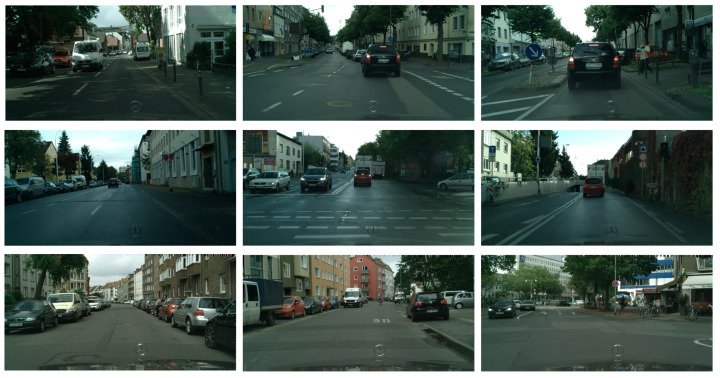
CityScapes dataset.

For this study, we selected part of the CityScapes dataset and divided the dataset into a training set, validation set, and test set according to the ratio of 7:1.5:1.5. Among them, the training set included 2334 images, the validation set included 500 images, and the test set included 500 images. The CityScapes dataset included 30 categories, considering the application scenario of this study was a traffic scenario; therefore, this study only detected 5 categories in the dataset, which were car, pedestrian, truck, bus, and rider. The CityScapes dataset is shown in Figure 10.

### 4.3. Evaluation Indicators

As a standard measure of the model metrics, the datasets in this paper were converted to the COCO dataset format. AP and mAP were used as metrics to measure the performance of target detection and instance segmentation of the model, respectively. In addition to AP and mAP, our metrics also included $AP_{50}$, $AP_{car}$, $AP_{person}$, $AP_{truck}$, $AP_{bus}$, and $AP_{rider}$. $AP_{50}$ represents the IOU threshold of 0.5, and $AP_{car}$, $AP_{truck}$, $AP_{bus}$, and $AP_{rider}$ represent the AP values for the five categories of car, pedestrian, truck, bus and rider, respectively. Such a standard setting can adequately evaluate the segmentation performance of the model.

### 4.4. Model Training and Experimental Parameter Configuration

The parameters of the model were set as follows: the number of samples selected for one training (batch size) was 4, the total number of training rounds was 30, and the total number of iterations was 22,200. The initial learning rate was set to 0.002 and the learning rate was divided by 10 at the 10th, 20th, and 25th epochs. To make the network training process more stable, the idea of migration learning was used to load the weight parameters from the Resnet network that had already been trained on the coco dataset to accelerate the training of the network.

**Figure 11:**
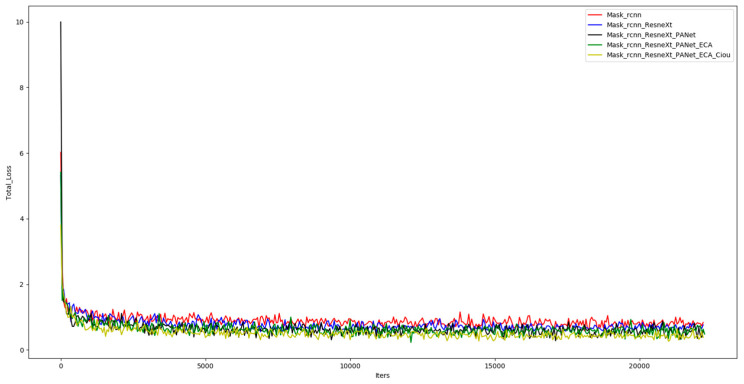
Loss function graph for each experimental group.

When training the improved Mask R-CNN model, the change curve of the loss function was plotted by the information of the result of each training round, as shown in Figure 11. As can be seen in the figure, the total loss values of the five groups of models converged at 22,200 training iterations. Compared with the other four groups of models, the loss function curve (yellow curve) of the improved Mask R-CNN model proposed in this paper significantly improved in convergence.

### 4.5. Ablation Experimental Results and Analysis

We conducted ablation experiments to explore the effects of the ResNeXt backbone network, the improved FPN module, the ECA attention module, and the CIoU loss function on model performance. The ablation experiments are shown in Table 1 and Table 2. Five groups of models were trained separately, and each module was added sequentially on the basis of Mask R-CNN, and the five groups of models were tested sequentially on the same test set.

Table 1 and Table 2 show the AP and mAP of the five groups of models for each category when IoU was taken as 0.5.

In Table 1 and Table 2, the improvement of the backbone network ResNeXt resulted in some improvement in the detection accuracy for all five types of targets. The improvement of detection accuracy for relatively small targets such as pedestrian and rider was more obvious, and the segmentation accuracy of these two types of targets was also significantly improved. The improved FPN structure showed that the detection accuracy for all five types of targets was significantly improved compared with the original model. It can be shown that in complex traffic scenes with more targets in front of the road, the improved FPN structure of the model can effectively fuse the low-level location information with the high-level semantic information, so that the denser multiple targets and small targets can be detected accurately. In terms of segmentation accuracy, the improved FPN structure resulted in a significant improvement of pedestrian, truck, and bus targets. The introduction of the ECA attention module slightly improved the detection accuracy of the model. It can be shown that ECA can effectively optimize the model performance and improve the detection accuracy of the model by optimizing the high-level low-resolution semantic information graph output from the backbone network, and it also has some improvement on the segmentation accuracy. Additionally, the use of the CIoU loss function for bounding box regression was able to optimize the model performance slightly. The detection accuracy for the truck targets was reduced, and the segmentation accuracy for the truck and rider targets was also slightly reduced. This situation was considered to be the result of the small number of truck and rider targets in the dataset, which in turn caused instability in the detection of such targets. Overall, the modules in our proposed improved Mask R-CNN algorithm effectively enhanced the recognition accuracy of the model. The detection accuracy was improved by 4.73%, and the segmentation accuracy was improved by 3.96% compared with the original Mask R-CNN algorithm, which verified the feasibility of the model.

For the Mask R-CNN model, we added improvement points individually to the network to perform experiments to evaluate the impact of each of its improvement points on the model in Table 3. Among them, the addition of the channel attention mechanism ECA had a minimal impact on the number of network parameters, but it helped a lot to improve the network. The addition of Ciou did not bring any change in the number of parameters, but it improved the accuracy by obtaining a more accurate bounding box. The group convolution of the ResNeXt network reduced the number of parameters and improved the performance of the model, while the FPN structure increased the computational complexity and the number of parameters to some extent, but it is crucial to improve the performance of the model.

**Figure 12:**
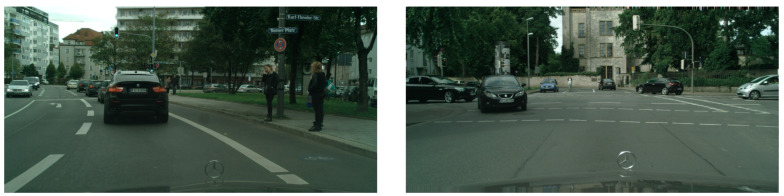
CityScapes test dataset.

**Figure 13:**
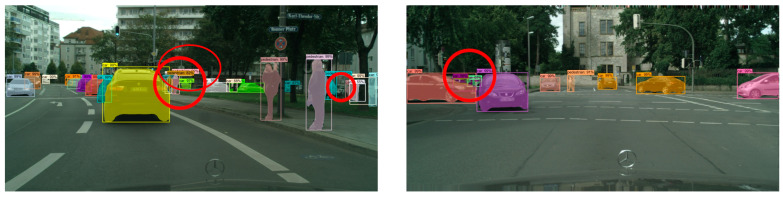
Test results of Mask R-CNN on CityScapes test dataset.

**Figure 14:**
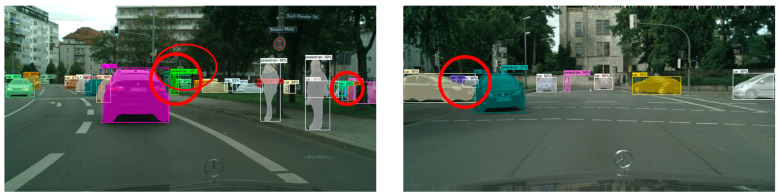
Improved Mask R-CNN test results on CityScapes test dataset.

In order to more intuitively evaluate the target detection and segmentation effects of the improved Mask R-CNN algorithm in different complex scenes, we selected the CityScapes test dataset to test five groups of models, and the experimental results are shown in Figure 12, Figure 13 and Figure 14.

Comparing the experimental results of the CityScapes test dataset, we found that in the original Mask R-CNN test results, although the model could detect different kinds of targets well, there were some false detections and poor mask quality. In the test results of the improved Mask R-CNN, it was able to detect different kinds of dense targets better. The mask segmentation results with good quality were also presented for the targets with serious occlusion. In the left experimental group, the dense targets in the middle and the right side of the picture had partial false detection. In the right experimental group, the masks of the two dense small targets on the left side of the picture were not of high quality, and although the targets were correctly identified as cars, the masks showed a confusing situation.

### 4.6. Migration Experiment Results and Analysis

**Figure 15:**
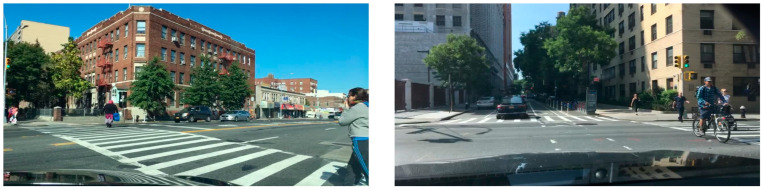
BDD test dataset.

**Figure 16:**
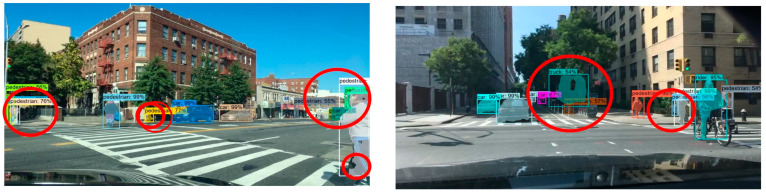
Mask R-CNN test results on BDD dataset.

**Figure 17:**
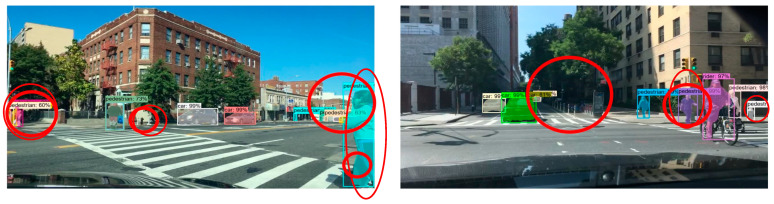
Improved Mask R-CNN test results on BDD dataset.

**Figure 18:**
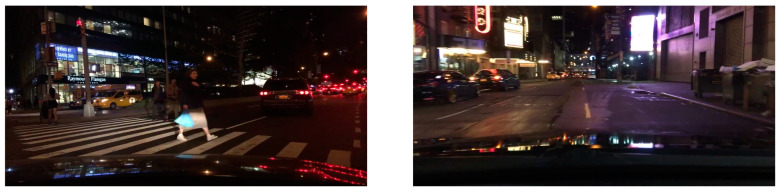
Dark scene images in BDD dataset.

**Figure 19:**
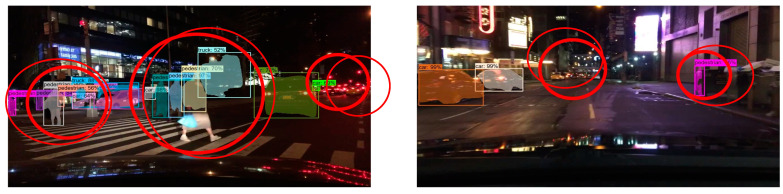
Mask R-CNN test results on dark scene images of BDD dataset.

**Figure 20:**
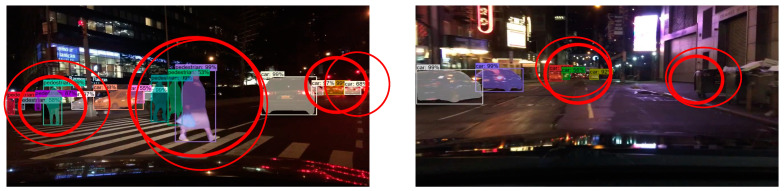
Improved Mask R-CNN test results on dark scene images of BDD dataset.

**Figure 21:**
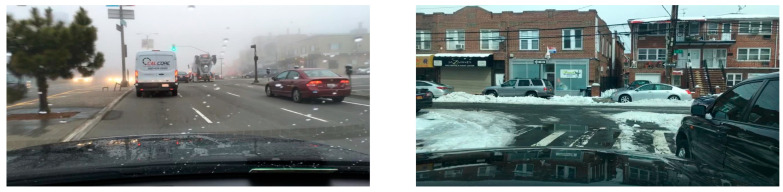
Rain and snow scene images for BDD dataset.

**Figure 22:**
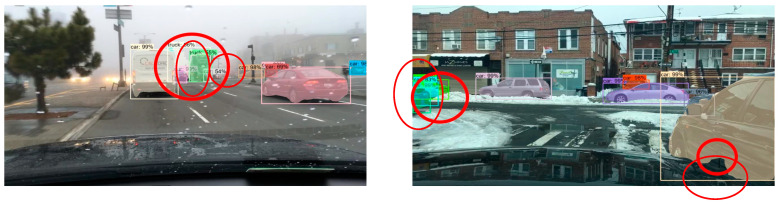
Mask R-CNN test results on rain and snow scene images of BDD dataset.

**Figure 23:**
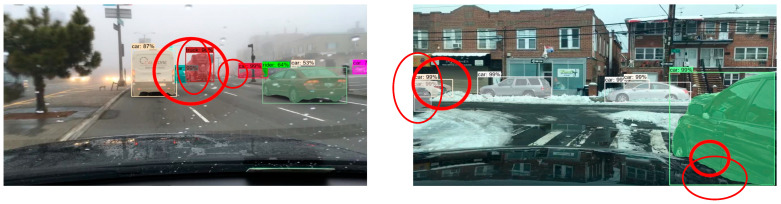
Improved Mask R-CNN test results on rain and snow scene images of BDD dataset.

In order to verify the generalization ability of the improved Mask R-CNN model, this subsection conducted migration experiments with the BDD autonomous driving dataset, which is a real-time image captured under driving conditions of real traffic scenes. The experiment was conducted with randomly selected sample images from different scenes in the dataset. The detection and segmentation results of the migration experiment are shown in Figure 15, Figure 16, Figure 17, Figure 18, Figure 19, Figure 20, Figure 21, Figure 22 and Figure 23.

In this group of experiments, the traffic scene pictures in the BDD dataset were selected as the test dataset. From the left experimental group, we can see that there were three false detections in the original Mask R-CNN test results. On the leftmost side of the image, the model detected two pedestrians as three pedestrians and the fence in front of the tree as a pedestrian. The right side of the picture also had the problem of poor-quality masking of the pedestrian. From the experimental group on the right, we can see that there were four false detections in the original Mask R-CNN test results: two pedestrian targets on the right side of the image, one car target in the middle of the image far away, and the truck target in the middle of the image. Moreover, comparing the two sets of experiments in this scene, it can be seen that the overall mask quality was higher in the improved Mask R-CNN test results, and the details of each target could be accurately segmented.

The detection and segmentation of targets in front of vehicles in dark scenes is difficult due to the influence of light. There may be a large number of false detections and missed detections in the recognition process, and the segmentation accuracy of the model is required to be very high. Therefore, in this set of experiments, we selected the images of darker night scenes in the BDD dataset for testing. Compared with the experimental results, we can see that there were more false detections and missed detections in the original Mask R-CNN test results, and the mask quality was not high as well. The improved Mask R-CNN could better detect multiple targets in front of the road in the dark scene, and the mask quality of each target was better.

While vehicle driving is affected by light, drivers also need to deal with various complex weather conditions. Therefore, in this set of experiments, we selected the images of rain and snow scenes from the BDD dataset for testing. Compared with the experiments on the left side, it can be seen that there were two false detections in the Mask R-CNN test results during rainy and snowy weather with a lot of fog in front. It was considered that the fog affected the feature extraction of the model for the front targets, which led to the low detection and segmentation accuracy. In the improved Mask R-CNN test results, the model could better detect all kinds of targets ahead. In the right experimental group, although the scene was clearer, the mask quality of each target was not high due to the influence of snow on the ground, and the details of each target could not be segmented better. The improved Mask R-CNN could accurately identify each target ahead and segment each target more completely.

The migration experimental results showed that the improved network model could still adapt and complete the detection and segmentation of multiple targets such as dense pedestrians and vehicles in various complex traffic scenarios for the new untrained sample dataset. It was proved that the proposed method had good migration performance, and the algorithm improvement was effective and feasible.

## 5. Conclusions

To address the problem of low detection and segmentation accuracy in the detection and segmentation of multiple targets in front of a vehicle in the environment perception of autonomous driving, this paper improved on the basis of Mask R-CNN. We improved the feature extraction capability of the backbone network by changing the backbone network ResNet to ResNeXt without increasing the complexity of the model. We also improved the FPN structure by adding a bottom-up path enhancement strategy. It can effectively shorten the information path and utilize the low-level accurate localization to improve the utilization of the low-level features of the network and facilitate the network to identify various targets more accurately. We also added the ECA attention module to the backbone feature extraction network to optimize the high-level, low resolution semantic information map output from the backbone network, which in turn led to a low resolution, high semantic feature information map for better identification of various types of dense medium and large targets. Finally, we replaced the loss function smooth L1 as CIoU for the two bounding box regressions in the model to improve the model convergence speed and optimize the network error. The experimental data showed that the loss function of the improved Mask R-CNN algorithm proposed in this paper had a smaller convergence value, and the mAP of target detection and instance segmentation were improved by 4.73% and 3.96%, respectively, compared with the original Mask R-CNN algorithm. The experimental results showed that the algorithm was more accurate and robust for the detection and segmentation of multiple targets ahead in traffic scenes. In the migration experimental test, the model could effectively identify various dense targets with good mask quality for different kinds of road traffic conditions and complex weather. In summary, the improved Mask R-CNN algorithm can be better applied to environment perception of autonomous driving with higher robustness and good generalization ability, which also has practical application prospects.

Although the improved Mask R-CNN proposed in this paper can better solve the problem of detecting and segmenting multiple targets in front of vehicles in complex scenes, there are still many areas to be improved in the future. For example, we will expand the number of targets in the dataset for road scenes and balance the number of targets in each category to avoid the problem of low target detection accuracy due to the small number of truck category samples in this study. The target detection and segmentation model proposed in this study will face a variety of traffic environments under diverse conditions in practical applications. The types of scenes provided by the dataset are still lacking compared to the actual situation. At the same time, it is still difficult to detect multiple targets in front of the vehicle under severe weather conditions, and the overall detection accuracy needs to be improved. Therefore, image quality assessment techniques will be introduced in future work to evaluate the quality of the dataset images. The low-quality images will be image-enhanced to improve the performance of the model. By continuously improving the detection and segmentation capabilities of the model, expanding the dataset, and supplementing with image quality assessment techniques, the future multi-objective recognition techniques for traffic scenes will be better applied to the environment perception of autonomous driving.

## Figures and Tables

**Table 1:** Ablation experiment: Target detection results.

	AP_car_	AP_pedestrian_	AP_truck_	AP_bus_	AP_rider_	mAP_det_
Mask R-CNN	72.21	54.71	42.11	63.78	56.62	57.89
Mask R-CNN + ResNeXt	73.81	55.36	43.95	65.82	58.66	59.52
Mask R-CNN + ResNeXt + Improved FPN	75.94	57.05	46.41	64.18	60.89	60.90
Mask R-CNN + ResNeXt + Improved FPN + ECA	75.63	59.86	47.06	66.86	60.75	62.03
Mask R-CNN + ResNeXt + Improved FPN + ECA + CIoU	75.79	60.82	46.68	68.16	61.65	62.62

**Table 2:** Ablation experiment: Instance segmentation results.

	AP_car_	AP_pedestrian_	AP_truck_	AP_bus_	AP_rider_	mAP_seg_
Mask R-CNN	66.41	46.08	42.31	60.57	52.74	53.62
Mask R-CNN + ResNeXt	68.42	49.17	43.15	60.24	54.55	55.11
Mask R-CNN + ResNeXt + Improved FPN	68.97	51.55	45.07	64.18	54.46	56.85
Mask R-CNN + ResNeXt + Improved FPN + ECA	68.91	54.36	45.32	63.48	55.48	57.51
Mask R-CNN + ResNeXt + Improved FPN + ECA + CIoU	69.14	54.17	44.74	65.20	54.67	57.58

**Table 3:** Comparison of model complexity.

	FPS/S^−1^	Parm/M	FLOPs/G	mAP_det/%	mAP_seg/%
Mask R-CNN	20.38	43.997043	194.4708	57.89	53.62
Mask R-CNN + ResNeXt	20.11	43.468915	197.6479	59.52	55.11
Mask R-CNN + FPN	19.85	46.461245	199.4276	58.66	55.96
Mask R-CNN + ECA	21.25	43.997064	194.5018	59.41	54.43
Mask R-CNN + CIoU	19.98	43.997043	194.4708	58.17	53.90

## Data Availability

Not applicable.

## References

[1] Grigorescu S., Trasnea B., Cocias T., Macesanu G. (2022). A survey of deep learning techniques for autonomous driving. J. Field Robot..

[2] Janai J., Güney F., Behl A., Geiger A. (2020). Computer vision for autonomous vehicles: Problems, datasets and state of the art. Found. Trends^®^ Comput. Graph. Vis..

[3] Su L., Sun Y.-X., Yuan S.-Z. (2022). A survey of instance segmentation research based on deep learning. CAAI Trans. Intell. Syst..

[4] Joseph R., Santosh D., Ross G., Ali F. You only look once: Unified, real-time object detection. Proceedings of the IEEE Conference on Computer Vision and Pattern Recognition.

[5] Liu W., Anguelov D., Erhan D., Szegedy C., Reed S., Fu C.-F., Berg A.C. (2016). Ssd: Single shot multibox detector. Proceedings of the Computer Vision–ECCV 2016: 14th European Conference.

[6] Girshick R., Donahue J., Darrell T., Malik J. Rich feature hierarchies for accurate object detection and semantic segmentation. Proceedings of the IEEE Conference on Computer Vision and Pattern Recognition.

[7] He K., Zhang X., Ren S., Sun J. (2015). Spatial pyramid pooling in deep convolutional networks for visual recognition. IEEE Trans. Pattern Anal. Mach. Intell..

[8] Girshick R. Fast r-cnn. Proceedings of the 2015 IEEE International Conference on Computer Vision (ICCV).

[9] Ren S., He K., Girshick R., Sun J. (2001). Faster r-cnn: Towards real-time object detection with region proposal networks. Advances in Neural Information Processing Systems.

[10] Bai M., Urtasun R. Deep watershed transform for instance segmentation. Proceedings of the IEEE Conference on Computer Vision And Pattern Recognition.

[11] Gao N.-Y., Shan Y., Wang Y., Zhao X., Yu Y., Yang M., Huang K. Ssap: Single-shot instance segmentation with affinity pyramid. Proceedings of the IEEE/CVF International Conference on Computer Vision.

[12] Dai J.-F., He K., Sun J. Instance-aware semantic segmentation via multi-task network cascades. Proceedings of the IEEE Conference on Computer Vision and Pattern Recognition.

[13] He K., Gkioxari G., Dollár P., Girshick R. Mask r-cnn. Proceedings of the IEEE International Conference on Computer Vision.

[14] Li Y., Qi H., Dai J., Ji X., Wei Y. Fully convolutional instance-aware semantic segmentation. Proceedings of the IEEE Conference on Computer Vision and Pattern Recognition.

[15] Bolya D., Zhou C., Xiao F., Lee Y.J. Yolact: Real-time instance segmentation. Proceedings of the IEEE/CVF International Conference on Computer Vision.

[16] Wang X., Kong T., Shen C., Jiang Y., Li L. (2020). Solo: Segmenting objects by locations. Proceedings of the Computer Vision—ECCV 2020: 16th European Conference.

[17] Ke L., Tai Y.-W., Tang C.-K. Deep occlusion-aware instance segmentation with overlapping bilayers. Proceedings of the IEEE/CVF Conference on Computer Vision and Pattern Recognition.

[18] Zhang T., Wei S., Ji S. E2ec: An end-to-end contour-based method for high-quality high-speed instance segmentation. Proceedings of the IEEE/CVF Conference on Computer Vision and Pattern Recognition.

[19] He J.-J., Li P., Geng Y., Xie X. (2023). FastInst: A Simple Query-Based Model for Real-Time Instance Segmentation. arXiv.

[20] Zhang H., Li F., Xu H., Huang S., Liu S., Ni L.M., Zhang L. (2023). MP-Former: Mask-Piloted Transformer for Image Segmentation. arXiv.

[21] Yurtsever E., Lambert J., Carballo A., Takeda K. (2020). A survey of autonomous driving: Common practices and emerging technologies. IEEE Access.

[22] Peng Y., Liu X., Shen C., Huang H., Zhao D., Cao H., Guo X. (2019). An improved optical flow algorithm based on mask-R-CNN and K-means for velocity calculation. Appl. Sci..

[23] He K., Zhang X., Ren S., Sun J. Deep residual learning for image recognition. Proceedings of the IEEE Conference on Computer Vision and Pattern Recognition.

[24] Lin T.Y., Dollár P., Girshick R., He K., Hariharan B., Belongie S. Feature pyramid networks for object detection. Proceedings of the IEEE Conference on Computer Vision and Pattern Recognition.

[25] Lu J.-H. (2021). Analysis and Comparison of Three Classical Color Image Interpolation Algorithms. J. Phys. Conf. Ser..

[26] Vinod N., Hinton G.E. Rectified linear units improve restricted boltzmann machines. Proceedings of the 27th International Conference on Machine Learning.

[27] Jonathan L., Shelhamer E., Darrell T. Fully convolutional networks for semantic segmentation. Proceedings of the IEEE Conference on Computer Vision and Pattern Recognition.

[28] Xie S., Girshick R., Dollár P., Tu Z., He K. Aggregated residual transformations for deep neural networks. Proceedings of the IEEE Conference on Computer Vision and Pattern Recognition.

[29] Liu S., Qi L., Qin H., Shi J., Jia J. Path aggregation network for instance segmentation. Proceedings of the IEEE Conference on Computer Vision and Pattern Recognition.

[30] Wang Q.-L., Wu B., Zhu P., Li P., Zuo W., Hu Q. ECA-Net: Efficient channel attention for deep convolutional neural networks. Proceedings of the IEEE/CVF Conference on Computer Vision and Pattern Recognition.

[31] Guo M.-H., Xu T.-X., Liu J.-J., Liu Z.-N., Jiang P.-T., Mu T.-J., Zhang S.-H., Martin R.R., Cheng M.-M., Hu S.-M. (2022). Attention mechanisms in computer vision: A survey. Comput. Vis. Media.

[32] Hu J., Li S., Sun G. Squeeze-and-excitation networks. Proceedings of the IEEE Conference on Computer Vision and Pattern Recognition.

[33] Zhang B., Fang S.-Q., Li Z.-X. (2021). Research on Surface Defect Detection of Rare-Earth Magnetic Materials Based on Improved SSD. Complexity.

[34] Zheng Z.-H., Wang P., Ren D., Liu W., Ye R., Hu Q., Zuo W. (2022). Enhancing geometric factors in model learning and inference for object detection and instance segmentation. IEEE Trans. Cybern..

[35] Lin T.-Y., Maire M., Belongie S., Bourdev L., Girshick R., Hays J., Perona P., Ramanan D., Zitnick C.L., Dollár P. (2014). Microsoft coco: Common objects in context. Proceedings of the Computer Vision–ECCV 2014: 13th European Conference.

[36] Cordts M., Omran M., Ramos S., Rehfeld T., Enzweiler M., Benenson R., Franke U., Roth S., Schiele B. The cityscapes dataset for semantic urban scene understanding. Proceedings of the 2016 IEEE Conference on Computer Vision and Pattern Recognition (CVPR).

